# Impact of Cryogenic Treatment on HCF and FCP Performance of β-Solution Treated Ti-6Al-4V ELI Biomaterial

**DOI:** 10.3390/ma13030500

**Published:** 2020-01-21

**Authors:** Anil Kumar Singla, Jagtar Singh, Vishal S. Sharma, Munish Kumar Gupta, Qinghua Song, Dariusz Rozumek, Grzegorz M. Krolczyk

**Affiliations:** 1 Department of Mechanical Engineering, Sant Longowal Institute of Engineering and Technology, Longowal 148106, Punjab, India; anilsingla@sliet.ac.in (A.K.S.); jagtarsingh@sliet.ac.in (J.S.); 2 Department of Industrial and Production Engineering, Dr B R Ambedkar National Institute of Technology, Jalandhar 144011, Punjab, India; sharmavs@nitj.ac.in; 3 Key Laboratory of High Efficiency and Clean Mechanical Manufacture, Ministry of Education, School of Mechanical Engineering, Shandong University, Jinan 250061, China; 4 National Demonstration Center for Experimental Mechanical Engineering Education, Shandong University, Jinan 250061, China; ssinghua@sdu.edu.cn; 5 Opole University of Technology, 76 Proszkowska St, 45-758 Opole, Poland; d.rozumek@po.opole.pl

**Keywords:** cryogenic, fatigue, titanium alloy, microstructure, crack propagation, fractography

## Abstract

The poor fatigue strength of Ti-6Al-4V ELI is a main cause of failure in structural implants. In this work, Ti-6Al-4V ELI was subjected to β-solution treatment to obtain martensite microstructure and further subjected to −196 °C for 24 h. Significant improvement in high cycle fatigue performance of martensite Ti-6Al-4V ELI was observed on exposure to cryogenic cycle. Resistance to fatigue crack growth of alloy was augmented in martensite structure as compared with mill annealed sample and the same was retained even after exposure to cryogenic treatment. The variation observed in fatigue behavior due to cryogenic treatment was correlated with fractography and metallurgical investigations. Improvement in high cycle fatigue performance can be attributed to a combined effect of a decrease in the size of prior β grain, formation of massive α patch and its subsequent transformation into ultra-fine α and β during the soaking period at −196 °C.

## 1. Introduction

Biomaterials as structural implants have been used to substitute the vanished or unhealthy biological structure to reinstate its shape and performance [1]. The authors observed the potential increase in the requirement of biomedical implants in particular for hip and knee joints after 2002. As per estimate by Kurtz et al. [2], the growth in demand of hip joints and knee joints is expected to be 174% and 673% respectively by 2030. Rack and Qazi [3] reported that due to limited life span of existing implants along with increase in human life expectancy, led to a rise in painful and undesirable revision surgeries. The present state of affair envisages the improvement in performance and longevity of structural implants, which commensurate with the human life span [1]. The authors highlighted that low fatigue strength is a major cause of failure in biomedical implants leading to revision surgery. There are many literature references focused on implant fatigue failure, in particular Chen and Thouas [4], premature failure in biomedical implants happened due to fatigue fracture and therefore biomaterials must have high fatigue strength and fracture resistance to ensure longevity of biomedical implants in the human body. Fatigue failure of biomedical implants due to variable and complex cyclic loading conditions has also been observed by Carrion et al. [5]. Renganathan et al. [6] advocated high fatigue strength of biomedical implants to prevent fracture failure under cyclic loading. Fatigue fracture is one of the primary reasons related to implant loosening resulting in failure [7]. Therefore, it is paramount that research should be focused on developing new biomaterials exhibiting better performance in terms of improved fatigue strength or fatigue properties of existing biomaterials must be enhanced by applying suitable material processing methods.

Titanium alloys are rapidly developing as first option for wider applications as reported by Geetha et al. [1]. Ti-6Al-4V is by far the most important (α + β) alloy, commonly known as the workhorse of the titanium industry as it encompasses 60% of titanium production [8]. It owns a typical combination of mechanical, physical and corrosion resistance properties, leading to its aggressive usage as a structural biomedical implant. Ren et al. [9] pointed out that poor fatigue resistance of titanium alloys leads to fatigue failure in biomedical implants. Magnissalis et al. [10] did investigations on the failure mechanism of two Ti-alloys HIP femoral stems. It was observed by authors that in both the cases main mechanism of fracture was fatigue. It was revealed that porous coating is a major factor for reduction in fatigue strength. Nayar et al. [11] conducted a failure analysis of five Ti-6Al-4V modular revision hip stems and concluded that in all five hip stems failure occurred due to fatigue mechanism. Chao and López [12] explored the mechanism of fracture failure in HIP prosthesis and have concluded that 90% of the fracture surface indicates fatigue failure. In view of excellent corrosion resistance, biocompatibility and very high strength to weight ratio of Ti-6Al-4V and its variants, it is worthwhile to explore the material processing techniques that can enhance its fatigue strength to ensure its longevity as an implant in the human body.

Cryogenic treatment (CT) is a well-known material processing method and has been adopted for attaining desirable mechanical properties of materials. Many review articles published in the literature at different times evidently endorse its effectiveness in augmenting the performance of ferrous materials. Cryogenic treatment has made significant contributions in improving wear resistance, tool life, dimensional integrity, hardness and product quality of cutting tools [13,14]. Baldissera and Delprete [15] highlighted fatigue strengthening of stainless steels due to formation of nano-martensite during the cryogenic treatment. According to research study [16], refinement of pearlite matrix and formation of fine martensite as important developments for enhancement of properties in low carbon steel and cast iron due to cryogenic treatment, while research study [17] reported compaction of crystal structure leads to much superior abrasive wear resistance, corrosion resistance and fatigue strength after cryoprocessing. Kalsi et al. [18] recommended a complete process of a thermal treatment in the following order: austenitization, quenching, cryogenic treatment and tempering. According to Gill et al. and Shokrani et al. [19,20], soaking temperature, soaking time and cooling/heating rate are important parameters of cryogenic treatment cycle and these parameters need to be optimized for different materials. While Singla et al. [21] have recommended soaking temperature in the range of −160 to −196 °C to realize the maximum improvement in the mechanical properties for materials. Soaking time of 24 h, cooling/heating rate of less than 2 °C/min have been recommended as optimum parameters. 

Cryogenic treatment has also been applied on non-ferrous materials and positive outcomes have been reported. According to Amini and Akhbarizadeh [22], corrosion and mechanical performance of the AZ91 magnesium alloy have improved after deep cryogenic treatment (DCT). Mohan et al. [23] noticed improvement in the performance of Al 7075 on exposure to cryogenic treatment. Li et al. [24] observed refinement in properties of Al alloys after DCT. Extensive research work has been published in the literature on CT of ferrous material but limited works have been reported in the literature pertaining to cryogenic treatment of non-ferrous materials. Review article [21] has done an exhaustive analysis of many research works on cryotreated non-ferrous materials and observed a tremendous potential for future research on CT of non-ferrous materials, including titanium alloys. Gu et al. [25] have investigated the influence of variable DCT on Ti-6Al-4V ELI and reported enhancement in tribological performance and hardness. However, these investigations were focused only on mill annealed (MA) Ti-6Al-4V ELI. According to research work by Singla et al. [26], cryogenic treatment has exhibited promising results in improving the wear behavior of the martensite Ti-6Al-4V ELI obtained by β-solution treatment, thereby increasing the possibility of its usage as biomedical implants. In this work, the effect of cryogenic treatment on high cycle fatigue (HCF) strength and fatigue crack propagation (FCP) resistance of martensite Ti-6Al-4V ELI is investigated to address its poor fatigue performance that hampers its applications in structural implants. 

## 2. Materials and Methods 

### 2.1. Materials

The chemical composition of Ti-6Al-4V ELI alloy as per American society for testing and materials (ASTM) standard [27] and as received material is given in Table 1. 

Monotonic quasi-static tension properties of MA Ti-6Al-4V ELI alloy as investigated are reported in Table 2.

### 2.2. Thermal Treatment

**Figure 1:**
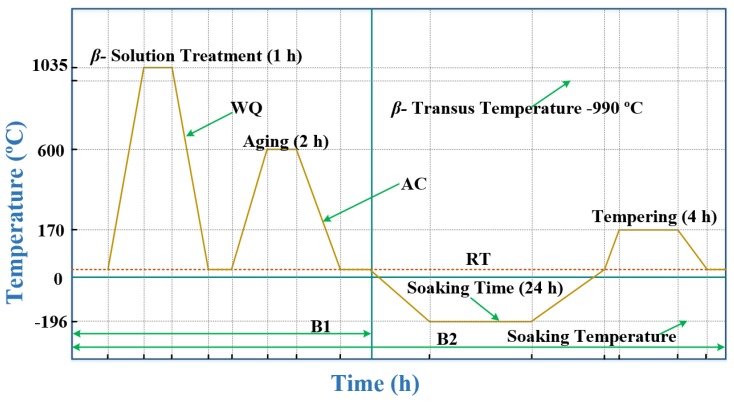
Schematic of thermal treatment schedules.

β-transus of Ti-6Al-4V ELI is found to be 990 °C and is determined based on the previous literature data [28,29,30,31,32]. Heat treatment above 990 °C and subsequent rapid cooling generates the martensite microstructure. The heat treatment (HT) as well as CT cycles considered in the present work are elucidated in Figure 1 for better understanding. Codes have been assigned to specimens of Ti-6Al-4V ELI for the purpose of presentation and discussions and the same is given in Table 3.

The samples of Ti-6Al-4V ELI have been exposed to ultrasonic cleaning in acetone to remove all external impurities. Ti-6Al-4V has a strong tendency to develop an oxygen affected zone at high temperature. The tendency to develop α-case formation and oxygen affected zone has been arrested by wrapping the specimens of B1 and B2 Ti-6Al-4V ELI in stainless steel (ss) 304 foil. The cleaned muffle furnace (Jupiter engineering works, New Delhi, India) was brought to 1035 °C before placing the samples inside the furnace. Specimens were held for 1 h and 10 min at 1035 °C in accordance with the procedure adopted by Imam et al. [33] to attain equilibrium conditions at furnace temperature in 10 min, and 1 h was used as holding time. HT specimens were allowed to attain 30 °C by quenching in 5% brine solution. HT was followed by aging at 600 °C for 2 h adopting the same procedure as done for heat treatment. These specimens were coded as B1 and B2.

Ultrasonic cleaning of specimens with acetone has been done at various stages of HT and sample preparation. The specimens coded as B2 were subjected to DCT using cryogenic processor CP220LH. Temperature of Ti-6Al-4V ELI specimens was lowered down from 30 to −196 °C slowly at a cooling rate of 1 °C/min. Specimens were soaked at that temperature for 24 h, and temperature of B2 specimens was raised to 30 °C slowly in increments of 1 °C /min. In the last, B2 samples were subjected to a tempering cycle (170 °C/4 h, air cooling—AC). 

### 2.3. High Cycle Fatigue and Fatigue Crack Propagation Test

An HCF test was performed using ASTM standard E466 on a rotating bending fatigue tester from (DUCOM, Bangalore, India). Investigations for HCF were conducted to determine the influence of CT post β-solution treatment on high cycle fatigue strength. Three numbers of specimens were tested for each thermal cycle. The parameters chosen for HCF test are given in Table 4. A weight of 6 kg was applied to generate a bending moment of 15.19 Nm leading to a stress amplitude of 604 MPa. Stress ratio R, ratio of minimum to maximum stress in each cycle, was −1. It signifies that compressive stress and tensile stress applied during the course of test is equal in magnitude. The number of cycles to failure was recorded and the average of three tests was reported.

FCP test has been conducted according to ASTM standard E647 using Nano Servo Hydraulic UTM from (Biss, ITW USA). Two holes separated by 14.05 mm (inter-centre distance) were used to hold the FCP specimen using clevises pins (diameter 6.35 mm). Crack opening displacement has been measured precisely by crack opening displacement (COD) clip gauge having a resolution of 1 µm. The test was done to assess the effect of CT post β-solution treatment on FCP behavior of Ti-6Al-4V ELI. The parameters chosen for FCP test are given in Table 5.

The research study [4] has recommended the load selection to be five times the body weight of a human being to simulate the actual reaction forces at joints during normal working. However, Dumbleton and Paul [34,35] have mentioned that effective load experienced by hip joint is 4.9 times the body weight and that by a knee joint is 2.8 times the body weight during normal functioning of the human being. Hence in this work a load of 4 kN was chosen that is equivalent to approximately five times the body weight of a human being. Wire EDM (EXCETEK S & T, Taichung city, Taiwan). was used to prepare the specimens for the FCP test. 

### 2.4. Microstructure and Fractography

Visualization of the microstructure and fractography was performed using JSM-6510 LV (JOEL, Tokyo, Japan) and SEM micrographs were analyzed using Image-J software (1.48v, Wayne Rasband, MD, USA). Silicon carbide papers of grade ranging from 100 to 3000 were used for wet grinding of the samples in aqueous media. A wet grinding procedure was adopted in order to avoid the undesirable changes in the microstructure due to overheating, which occurs due to low thermal conductivity (6.6 W/mK) of T-6Al-4V ELI alloy. At the same time, aqueous media is effective in removal of loose particles of metal and abrasives as well as for keeping the specimen cool leading to minimization of the grinding defects. The mechanical polishing of specimens was accomplished using velvet cloth laden with the suspension of abrasive containing Al_2_O_3_ powder and distilled water. The procedure was repeatedly done to ensure better specimen preparation. Kroll’s etchant containing 3 mL HF, 6 mL HNO_3_ and 91 mL distilled water was used as etching agent and etching was done through immersion as reported in the literature [28,36]. Specimens were slowly rotated while immersed in etchant for 10–12 s in order to ensure uniform application of etchant. Specimens were observed for obtaining SEM images.

A layer of 5 mm depth of fractured surface of HCF specimens of Ti-6Al-4V ELI alloy was obtained by wire EDM in order to examine the fracture during HCF testing. Crack path of FCP specimens was analyzed using SEM fractography.

## 3. Results and Discussions

### 3.1. Microstructure

#### Microstructure of MA, B1 and B2 Ti-6Al-4V ELI 

**Figure 2:**
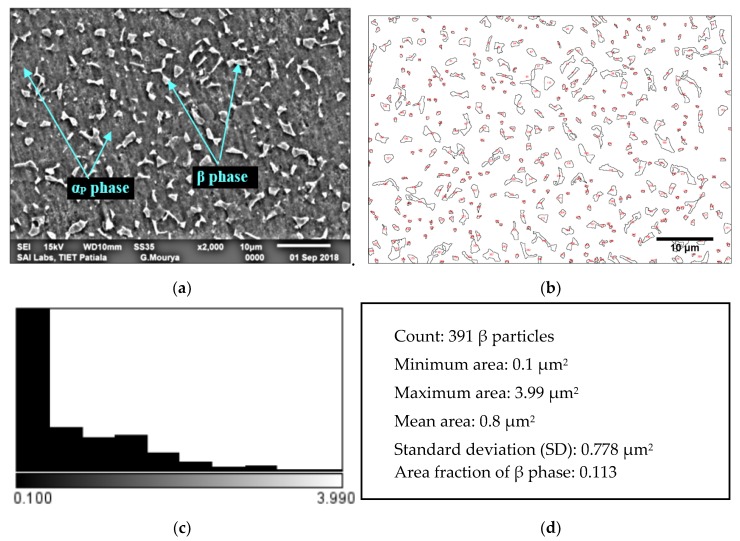
Microstructure of (**a**) MA Ti-6Al-4V ELI; (**b**) threshold image of MA Ti-6Al-4V ELI; (**c**) β phase distribution and (**d**) statistical analysis of β phase.

The microstructure of MA Ti-6Al-4V ELI alloy is shown in Figure 2a, which is characterized by the presence of β phase (globular particles) in the matrix of primary α (α_P_). The microstructure of the MA specimen is in agreement with that reported by Gu et al. [37]. Statistical analysis of β phase has been done using ImageJ software. Threshold image of MA Ti-6Al-4V ELI is shown in Figure 2b and β phase distribution is presented in Figure 2c. The details of statistical analysis of β phase observed in MA Ti-6Al-4V ELI is reported in Figure 2d.

**Figure 3:**
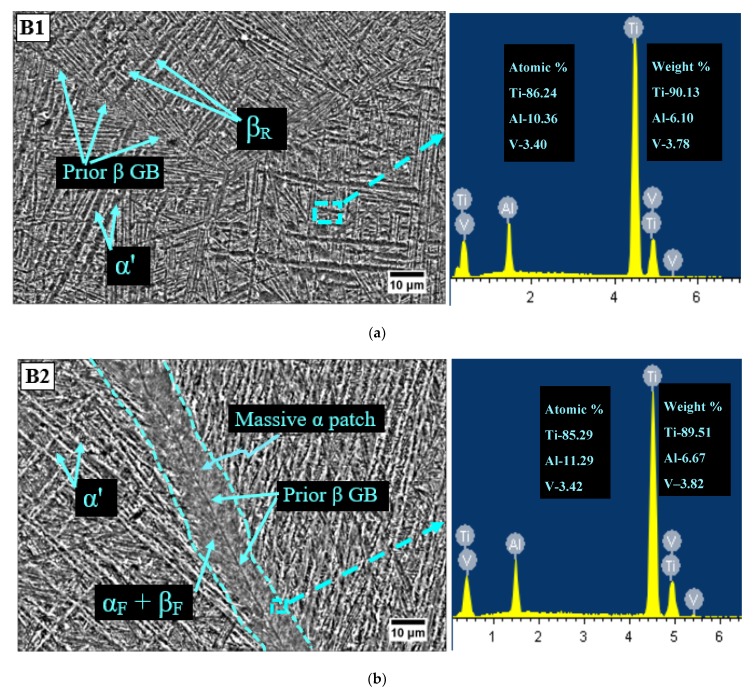
Microstructure of Ti-6Al-4V ELI for (**a**) the B1 specimen (β-solution treatment (1035 °C/1 h, water quenching (WQ)) + aging (600 °C/2 h, air cooling (AC))) and (**b**) the B2 specimen (β-solution treatment (1035 °C/1 h, WQ) + aging (600 °C/2 h, AC) + deep cryogenic treatment (DCT; −196 °C/24 h) + tempering (170 °C/4 h, AC)).

After HT at 1035 °C for a 1 h holding period, microstructures of B1 and B2 specimens were having an unstable β phase. Transformation of β phase to martensite α (α’) occurred due to the suppression of a diffusion-controlled process after quenching in 5% brine solution from 1035 °C. Martensite alpha consisted of α’ having needle like morphology, orthogonally oriented within the prior β grain as shown in Figure 3a. The α’ phase was reported to be rich in vanadium [38] and is further substantiated from the energy dispersive X-ray spectroscopy (EDS) graph of the B1 specimen shown in Figure 3a. The martensite finish temperature for Ti-6Al-4V ELI is below 30 °C (room temperature—RT) [28]. Therefore, untransformed β, designated as retained β (β_R_) was also there in the B1 sample. The different microstructural elements such as α’, β_R_ and grain boundary of prior β were clearly detected in the SEM image of specimen B1 Figure 3a. The microstructure of the B2 specimen of Ti-6Al-4V ELI is shown in Figure 3b. The cryogenic cycle caused massive α development that was further transformed into ultrafine α(α_F_) and β(β_F_) precipitates during the soaking phase at −196 °C. The presence of (α_F_) and β(β_F_) precipitates was endorsed from the EDS graph of B2 shown in Figure 3b.

The formation of massive alpha initiated from the grain boundaries of prior β and further propagated into the corresponding grains. The morphological analysis reflects the same response for massive α as per the previous literature works [39,40]. As the cryogenic cycle exposed the B2 samples to a temperature below M_f_, which led to the decomposition of β_R_ into fine α and β. The width of massive α was observed at five distinction points, and recorded mean width was 14.05 µm. The mean sizes of prior β grain for B1 and B2 specimens were 456 µm and 408 µm, respectively. The observed decline in size of prior β grain by 10.52% was due to cryogenic treatment of B2 specimen.

### 3.2. High Cycle Fatigue

**Figure 4:**
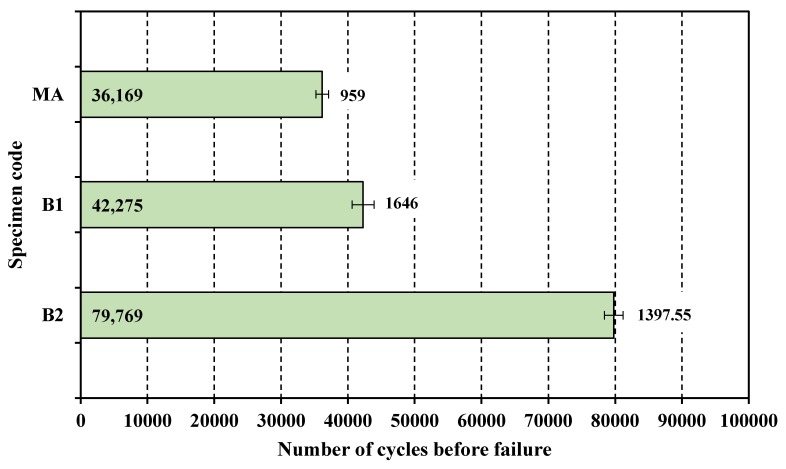
HCF mean results and standard deviation (for data dispersion) of B1, B2 and MA Ti-6Al-4V ELI.

The HCF test was conducted on three samples for each category of specimen using test parameters given in Table 3. The average of three tests was computed and was plotted as a bar chart in Figure 4. The HCF mean results along with standard deviation (for data dispersion) is reflected in Figure 4. It is clear from the results that martensite Ti-6Al-4V ELI had augmented the HCF performance as reflected from the increase in the number of cycles before failure. The percentage improvement in B1 specimen in comparison with MA specimen was 16.88%. Cryogenic treatment further increased the HCF performance of martensite specimen (B2) by 88.69%. 

**Figure 5:**
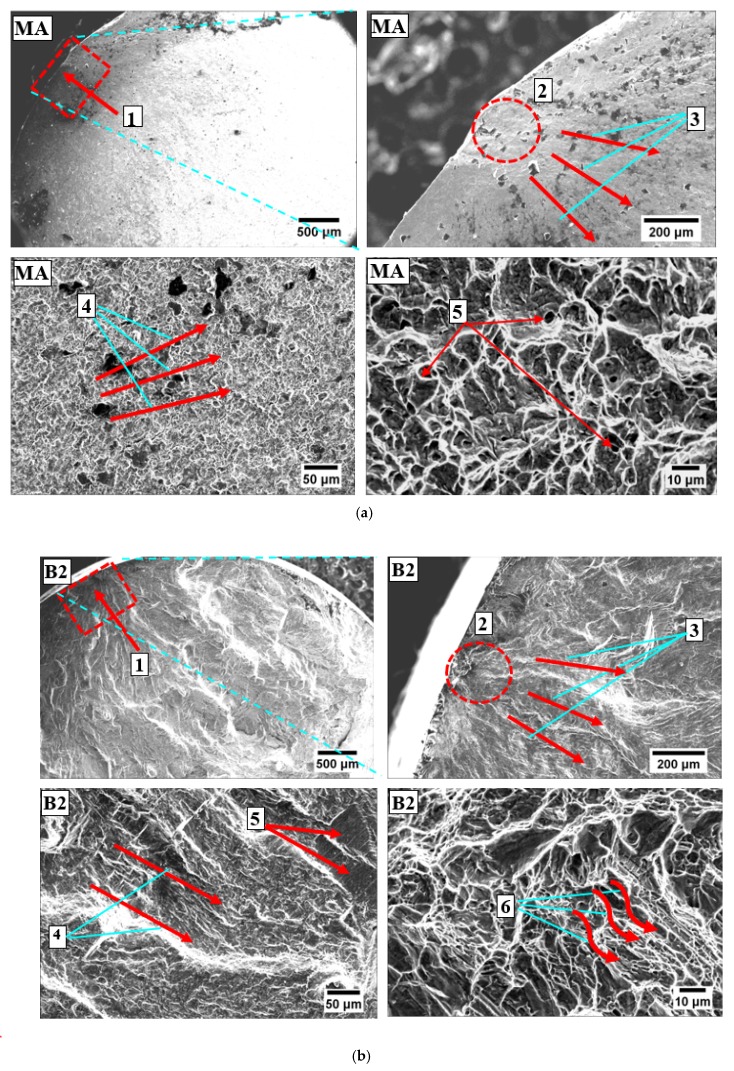
SEM fractography images of Ti-6Al-4V ELI for (**a**) MA, note: 1—crack initiation point, 2—crack initiation, 3—slow crack propagation, 4—fast crack propagation, 5—fine equiaxed dimples and (**b**) B2 (β-solution treatment (1035 °C/1 h, WQ) + aging (600 °C/2 h, AC) + DCT (−196 °C/24 h) + tempering (170 °C/4 h, AC)), note: 1—crack initiation point, 2—crack initiation, 3—slow crack propagation, 4—fast crack propagation, 5—facets, 6—striations.

The improvement in the HCF property, reflected in terms of the increased number of cycles to failure, had happened due to a combined effect of a decrease in the size of prior β grain, formation of massive α patch and its subsequent transformation into ultra-fine α and β during the soaking period at −196 °C. Improvement in HCF performance with a reduction in the size of the prior β grain has also been reported earlier in different research studies [41,42,43]. Fractography using scanning electron microscopy was done to have further insight into the nature of failure and analysis of fractured surface. SEM fractography of MA and B2 specimens was presented in Figure 5a,b respectively. Fractured surfaces were examined for prime features viz. crack initiation—2 including crack initiation point—1, crack growth behavior (3—slow crack propagation, 4—fast crack propagation see Figure 5) and nature of the fracture. Figure 5a has also shown fine equiaxed dimples—5. In the fractographic image of the MA specimen, crack initiation as well as crack propagation was quite smooth. The fractured surface was marked by fine equiaxed dimples indicating a ductile fracture. The image of the fractured surface of B2 samples was marked by tortuous crack propagation. The fractured surface was having facets—5 and striations pointing—6 towards mixed mode of fracture (see Figure 5b). The most distinguishing feature of the fractographic image of the B2 specimen was a tortuous crack path primarily responsible for an increase in the number of cycles to failure. 

### 3.3. Fatigue Crack Propagation

**Figure 6:**
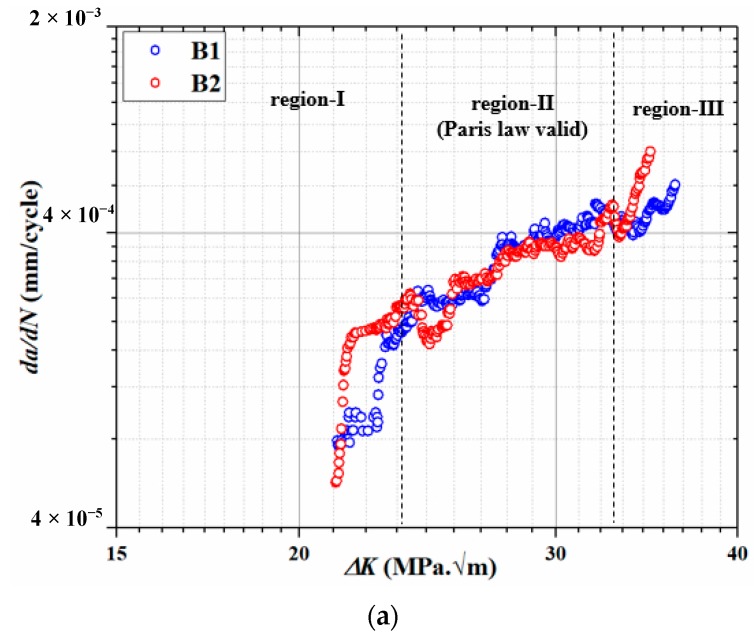

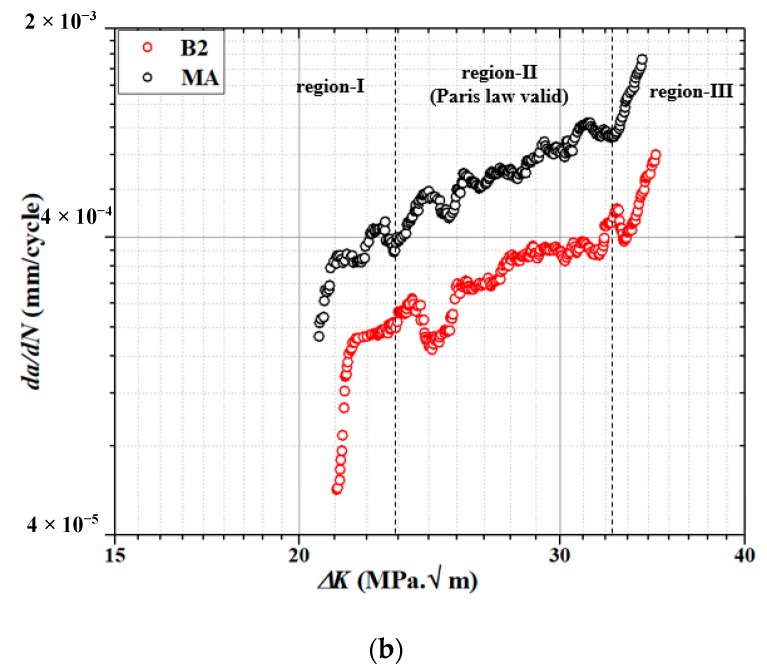
*da/dN-ΔK* curves of Ti-6Al-4V ELI for (**a**) B1 and B2, and(**b**) B2 and MA.

A fatigue crack propagation (FCP) test was performed using the parameters given in Table 4 for each category of specimens of Ti-6Al-4V ELI alloy. The FCP data was continuously measured using the COD clip gauge. The fatigue crack growth rate, i.e., *da/dN* was plotted against a stress intensity factor, i.e., *ΔK* for different microstructures. To visualize the effect of CT on the martensite Ti-6Al-4V ELI, *da/dN* was plotted against *ΔK* for B1 and B2 specimens as shown in Figure 6a. There are three regions of the plot namely region-I, region-II and region-III. Region-I signifies the crack initiation phase, region-II represents the segment of the plot where Paris law is valid and region-III indicates an unstable fracture. It is clear from the plot shown in Figure 6a that crack initiations in B1 and B2 specimens were almost identical. The FCP rate was quite similar for B1 and B2 specimens in the region-II (Paris validity), which was primarily the most important region in characterizing FCP properties. FCP behavior of B2 specimen was on lower side as compared to B1 sample in region-III. The minor decline in the FCP behavior of the martensite microstructure after exposure to cryogenic temperatures could be attributed to the refinement of the prior β grain. In order to assess the improvement in the FCP properties of B2 specimen versus MA sample, *da/dN* was plotted against *ΔK* for MA and B2 samples as shown in Figure 6b. It is distinct from the plot that B2 specimen had displayed significantly improved FCP resistance in all the three regions.

**Figure 7:**
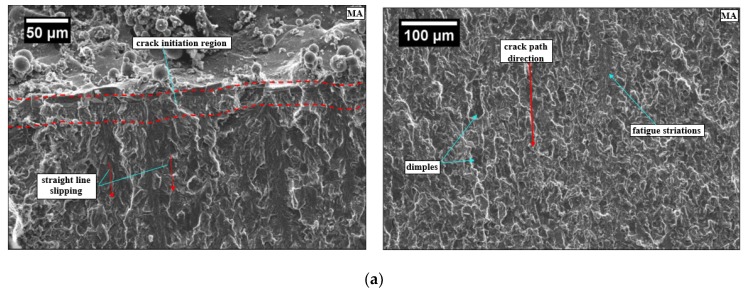

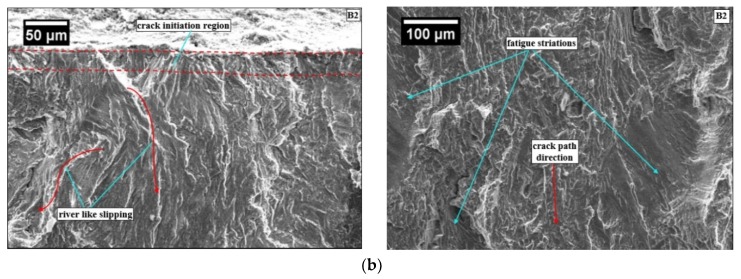
SEM fractography image of Ti-6Al-4V ELI for (**a**) MA FCP specimen and (**b**) B2 FCP specimen.

For further insight into the FCP behavior, SEM images (Figure 7) depict the crack propagation path in MA and B2 samples. There are no bifurcations visible in the crack path in MA specimen leading to smooth crack growth once initiated. It is clear from straight line slipping shown in Figure 7a. The results are in complete agreement with earlier research studies [41,44]. The crack path followed by B2 specimen is quite tortuous due to deflections caused by prior β grain boundaries and orthogonal needle like martensite α’. The random orientation of martensite α’ within prior β grain also leads to the generation of secondary cracks reflected in terms of river like slipping as shown in Figure 7b. Bifurcations of macrocracks into secondary microcracks and severe deflections of cracks in β heat treated Ti-6Al-4V has also been reported by earlier research studies [41,43,44]. The net effect of crack deflection is to disperse the strain field energy of the macroscopic crack into multiple secondary microcracks resulting in a significant reduction in fatigue crack growth [44].

## 4. Conclusions

This work investigated the influence of CT on HCF and FCP performance of martensite and mill annealed microstructure of Ti-6Al-4V ELI alloy. The following conclusions were drawn.

1. Cryogenic treatment had caused refinement in the prior β grain and led to development of massive α and its further transformation into ultrafine α and β during the soaking phase at cryogenic temperature.
2. CT had caused improvement (88.69%) in HCF behavior of martensite microstructure attributable to refinement in the prior β grain and development of massive α that was further transformed into ultrafine α and β during the soaking phase at cryogenic temperature.
3. Significant improvement in fatigue crack propagation was observed after β-solution treatment due to formation of martensite α’ and prior β grains. Cryogenic treatment post β-solution treatment had not realized any improvement in the FCP behavior of martensite Ti-6Al-4V ELI. However, despite prior β grain reduction after CT, no decline in the FCP performance of martensite Ti-6Al-4V ELI was noticed.
4. Cryotreated martensite specimen had displayed significantly improved FCP behavior when compared with MA sample in all the three regions of *da/dN* vs. *ΔK* plot attributable to crack tortuosity. Crack deflection is to disperse the strain field energy of the macroscopic crack into multiple secondary microcracks resulting in significant reduction in fatigue crack growth.

In view of these inferences it could be concluded that the cryogenic cycle had significantly improved the HCF performance and cryo-treated martensite Ti-6Al-4V ELI could be recommended for use in structural biomedical implants. However, it will be interesting to investigate the effect of higher tempering temperature (up to 600 °C) and a longer holding time (up to 24 h) during the tempering cycle post cryogenic treatment on HCF and FCP performance of the Ti-6Al-4V ELI alloy.

## Figures and Tables

**Table 1:** Chemical composition (wt %) of Ti-6Al-4V ELI alloy.

Ti-6Al-4V ELI	Fe	C	N	H	O	Al	V	Ti
As per ASTM F136-13	≤0.25	≤0.08	≤0.05	≤0.012	0.13	5.5–6.5	3.5–4.5	balance
As received material	0.04	0.02	0.01	0.001	0.10	5.9	4.2	balance

**Table 2:** Monotonic quasi-static tension properties of mill annealed (MA) Ti-6Al-4V ELI alloy.

Material	σ_y_ (MPa)	σ_u_ (MPa)	A (%)	HRC
MA Ti-6Al-4V ELI	958	1040	19.6	32

**Table 3:** Details of thermal cycle.

Thermal Cycle	Specimen Code
β-solution treatment (1035 °C/1 h, WQ) + aging (600 °C/2 h, AC)	B1
β-solution treatment (1035 °C/1 h, WQ) + aging (600 °C/2 h, AC) + DCT(−196 °C/24 h) + tempering (170 °C/4 h, AC)	B2
mill annealed (MA)	MA

**Table 4:** Parameters used in high cycle fatigue (HCF) test.

Parameter	Value of the Parameter
Stress ratio R	−1
Stress amplitude	604 MPa
Frequency	20 Hz

**Table 5:** Parameters used in fatigue crack propagation (FCP) test.

Parameters of FCP Test	Value of the Parameter
Load	4 kN
Frequency	10 Hz
Stress ratio R	0.1

## Nomenclature and Abbreviation

*da/dN*	fatigue crack growth rate
σ_y_	yield strength in quasi-static tension
σ_u_	ultimate tensile strength in quasi-static tension
A	% elongation
R	stress ratio
M_f_	martensite finish temperature
*ΔK*	stress intensity factor range
AC	air cooling
COD	crack opening displacement
CT	cryogenic treatment
DCT	deep cryogenic treatment
EDS	energy dispersive X-ray spectroscopy
FCP	fatigue crack propagation
HCF	high cycle fatigue
HT	heat treatment
MA	mill annealed
RT	room temperature
SEM	scanning electron microscope
WQ	water quenching
ASTM	American society for testing and materials
